# Alternative splicing of the *FLOWERING LOCUS C*-like gene *MaMADS33* is associated with endodormancy in mulberry

**DOI:** 10.48130/forres-0024-0027

**Published:** 2024-09-04

**Authors:** Yiwei Luo, Hongjiang Liu, Yuanxiang Han, Wei Li, Wuqi Wei, Ningjia He

**Affiliations:** State Key Laboratory of Resource Insects, Southwest University, Chongqing 400716, China

**Keywords:** *FLOWERING LOCUS C*, Mulberry, Alternative splicing, Endodormancy

## Abstract

Alternative splicing (AS) is an important post-transcriptional process that generates multiple mRNA isoforms. *FLOWERING LOCUS C* (*FLC*) is a pivotal gene in both the vernalization and autonomous pathways of flowering plants, and *MaMADS33* is one of the *FLC* homologs in white mulberry (*Morus alba*). Recent studies have revealed that *MaMADS33* is involved in endodormancy, but the underlying molecular mechanism remains to be characterized. Here, a comparison of *MaMADS33* expression among three mulberry cultivars with different degrees of dormancy revealed a positive association between *MaMADS33* expression and dormancy. Further 3' and 5' rapid amplification of cDNA ends (RACE) analyses led to identifying four *MaMADS33* isoforms derived from AS and designated *MaMADS33-AS1*–*4*. Analysis of their coding potential revealed that *MaMADS33-AS1* was a long non-coding RNA. Expression profiling and splicing-efficiency analyses showed that cold stress during endodormancy induced AS of *MaMADS33*, resulting in a predominance of truncated isoforms, especially *MaMADS33-AS1*. *MaMADS33-AS2* expression was upregulated during both endodormancy and ecodormancy, whereas *MaMADS33-AS3* and *MaMADS33-AS4* were endodormancy-associated isoforms that were upregulated during endodormancy and then downregulated during ecodormancy. MaMADS33-AS4 was used as bait for a yeast two-hybrid screen because its gene expression was higher than that of MaMADS33-AS3, and mulberry winter-accumulating 18 kDa protein (MaWAP18) was identified as an MaMADS33-AS4 interaction partner. The interaction between MaWAP18 and MaMADS33-AS4 was confirmed by a bimolecular fluorescence complementation assay. These findings offer insight into the role of *FLC* homologs in the endodormancy of woody plants.

## Introduction

Bud dormancy of perennials is a survival strategy for adaptation to the strikingly different environmental conditions of summer and winter^[1]^. Based on the specific conditions that trigger dormancy, it can be divided into endodormancy, aradormancy, and ecodormancy^[2]^. Endodormancy is the deep dormancy of plants that is established through low temperatures or short photoperiods in autumn and winter, and the release of endodormancy requires a specific period of chilling. Aradormancy refers to the dormancy of lateral buds that results from apical dominance, whereas ecodormancy refers to a temporary halt in growth caused by adverse environmental conditions^[2]^. Endodormancy is intimately linked to fruit set and quality, as a lack of sufficient chilling exposure can lead to abortion of reproductive whorls, low bud burst, and non-uniform bloom^[3−5]^. Given the ongoing trend of global warming, the mechanism of endodormancy has garnered increasing attention^[6−9]^.

Low temperatures and short photoperiods are two crucial factors required for the induction of endodormancy in plants^[10]^, but the underlying mechanisms are not fully understood. For a long time, research on endodormancy in woody plants has concentrated primarily on external phenotypic manifestations and biochemical components. The first dormancy-associated *MADS-box* (*DAM*) genes were identified through localization and cloning of the evergrowing locus in peach^[11,12]^. This discovery significantly expedited the analysis of the molecular mechanisms underlying endodormancy. The *DAM* genes are homologs of the flowering genes *SHORT VEGETATIVE PHASE* (*SVP*) and *AGAMOUS LIKE24* (*AGL24*) in *Arabidopsis*^[13,14]^. In recent years, the involvement of the *DAM* genes in the endodormancy process of perennial plants has been confirmed at the nucleic acid level^[15−20]^. Furthermore, other flowering genes, including *FLOWERING LOCUS T*/*TERMINAL FLOWER 1* (*FT*/*TFL1*)^[21,22]^ and *SUPPRESSOR OF OVEREXPRESSION OF CONSTANS1* (*SOC1*)^[23]^, as well as regulatory genes associated with plant hormones such as ABA and GA^[24,25]^, also play pivotal roles in regulating the dormancy of woody plants.

The MADS-box family gene *FLOWERING LOCUS C* (*FLC*) serves as the central integrator of the vernalization pathway in *Arabidopsis*^[26]^. Numerous studies have demonstrated that *FLC* also plays an important role in the regulation of endodormancy in perennial plants. In *Poncirus trifoliata*, expression of *PtFLC* is upregulated during autumn and winter but downregulated in spring, coinciding with the release from dormancy^[27]^. Similarly, two *FLC* homologs in apple (*Malus domestica*), *MdMADS135*, and *MdMADS136*, exhibit expression patterns comparable to those of *PtFLC*^[28]^. Notably, the expression of *MdoFLC* is strongly correlated with endodormancy, and its chromosome position is linked to the timing of bud break^[29,30]^. Ectopic expression in *Arabidopsis* revealed that *MdoFLC* functions as a growth inhibitory factor during endodormancy^[31]^. Transcriptome studies in buds of kiwifruit (*Actinidia chinensis*) during endodormancy showed that *AcFLCL* expression peaks during the accumulation of chilling hours, and overexpression of *AcFLCL* promotes budbreak^[32]^. However, the precise role and mechanism by which FLC homologs regulate endodormancy in perennial plants remain to be clarified.

Alternative splicing (AS) is a post-transcriptional process that generates multiple mRNA isoforms through seven major types of splicing events^[33,34]^. AS plays major roles in responses to environmental cues, including cold temperatures^[35−37]^. Research in pear (*Pyrus pyrifolia*) showed that AS of *DAM* genes was involved in dormancy^[38]^. Similarly, two sense AS isoforms of *AcFLCL* have been identified in kiwifruit, with the longer isoform induced by chilling and the shorter isoform detected before, and after chilling treatment^[32]^.

White mulberry (*Morus alba*) is a perennial woody plant whose fruit is rich in anthocyanins and has numerous medicinal and health benefits^[39]^. A chilling period is essential for breaking endodormancy in mulberry^[40]^. Mulberry sclerotial disease, a widespread fungal infection, poses a significant threat to the production of mulberry^[41]^; it typically erupts from January to April, peaking in mid-March^[42]^. Staggering the flowering times of mulberry trees to avoid this infection period could be an effective strategy for disease control.

Our previous study demonstrated that the mulberry *FLC* homolog *MnMADS33* is upregulated by cold treatment under field and artificially controlled conditions, and its downregulation is associated with the release from endodormancy^[43]^. In this study, the role of *MaMADS33* in mulberry endodormancy was further investigated. Comparative expression analysis revealed a positive association between *MaMADS33* expression and mulberry endodormancy. Using 3' RACE and 5' RACE techniques, we identified four splicing isoforms of *MaMADS33* during endodormancy. Cloning and expression analyses showed that cold stress during endodormancy induced AS of the *MaMADS33* gene, resulting in a predominance of truncated isoforms, especially the long noncoding RNA *MaMADS33-AS1*. Expression profiling indicated that *MaMADS33-AS4* expression was positively associated with mulberry endodormancy. Furthermore, winter-accumulating 18 kDa protein (MaWAP18) was identified as a MaMADS33-AS4 interaction partner through yeast two-hybrid screening and bimolecular fluorescence complementation (BiFC) assays. Collectively, the present findings suggest that *MaMADS33* plays a crucial role as an endodormancy regulator in mulberry.

## Materials and methods

### Plant materials

Flower buds were collected from the diploid *M. alba* varieties 'Jinqiang63' (JQ63), 'Lunjiao109' (LJ109), and 'Zhenzhubai' (ZZB) growing at the National Mulberry Breed Improvement Center of the Southwest University of China (29°49'18"N, 106°25'45"E). These distinct mulberry varieties hail from various geographic regions within China. LJ109 was sourced from Foshan City in Guangdong Province, JQ63 is a local cultivar from Dazu District in Chongqing, and ZZB originated in Linqing City in Shandong Province. The flower buds of LJ109, JQ63, and ZZB break in January, early February, and late February, respectively, demonstrating the progressive increase in dormancy duration of these three varieties.

For comparative expression analysis, flower buds of LJ109, JQ63, and ZZB were collected at six time points from autumn through spring: 16 October 2017, 6 November 2017, 27 November 2017, 21 December 2017, 12 January 2018, and 25 February 2018. For other analyses, flower buds of JQ63 were collected at five time points: 23 October 2020, 26 November 2020, 25 December 2020, 23 January 2021, and 24 February 2021. At each time point, buds were harvested from three replicate groups, each consisting of three to four independent plants of the same genotype. Before molecular analyses, the buds were processed by removing their scales, immediately freezing them in liquid nitrogen, and storing them at −80°C. Daily maximum temperature data were retrieved from a reliable online source (http://lishi.tianqi.com/).

### Quantitative Real–Time PCR (qRT–PCR) and RT–PCR analyses

Flower buds frozen at −80 °C were ground in liquid nitrogen in an RNA-free environment. Total RNA was isolated using the TRIzol Reagent (Invitrogen, Carlsbad, CA, USA) according to the manufacturer's instructions. First-strand complementary DNA (cDNA) was synthesized using a PrimeScript RT reagent Kit with gDNA Eraser (Perfect Real Time) (Takara, Dalian, China), followed by second-strand cDNA synthesis. qRT–PCR was performed in a volume of 20 μL using the ABI7500 Fast Real-time PCR system (Applied Biosystems, Foster City, CA, USA) and SYBR Green I Master Mix (Takara, Dalian, China). The mulberry *MaRPL15* gene (Morus024083) served as the internal control^[44]^. Relative gene expression was calculated using the formula 2^−[Ct(target gene) − Ct(control gene)]^. RT–PCR was performed using primers specific to the open reading frame (ORF) of each AS isoform. PCR products were run on 1% agarose gels and verified by sequencing. The primers used for qRT–PCR and RT–PCR are listed in Supplemental Table S1.

### 3' and 5' rapid amplification of cDNA ends (RACE)

*MaMADS33* transcripts were amplified by 3' RACE and 5' RACE using the 3'-Full RACE Core Set with PrimeScript RTase (Takara, Dalian, China) and the FirstChoice RLM-RACE Kit (Invitrogen Life Technologies, Carlsbad, CA, USA), respectively, according to the manufacturer's instructions. The template used for these amplifications was a cDNA pool derived from flower buds of JQ63 collected on 23 October 2020, 26 November 2020, 25 December 2020, 23 January 2021, and 24 February 2021. The primers are listed in Supplemental Table S1. The gene structure of *MaMADS33* was visualized using the Gene Structure Display Server 2.0 (GSDS) with the *Morus notabilis* genome^[45]^. A multiple alignment of the MaMADS33-AS2, MaMADS33-AS3, and MaMADS33-AS4 protein sequences were obtained using ClustalW^[46]^.

### Definition of mulberry endodormancy

Mulberry endodormancy was defined as described by Walton^[47,48]^. In brief, buds were considered to be endodormant if fewer than 50% of them reached the green tip stage after exposure to suitable environmental conditions. Shoot cuttings with two buds were immersed in distilled water at room temperature (22–25 °C), and the water was changed every 2 d. The bud-break percentage was recorded after four weeks.

### Splicing efficiency assay

Splicing efficiency was measured as described previously^[49]^. Unspliced primers were designed to span intron–exon junctions, and spliced primers were designed to cross exon–exon junctions. All PCR products were verified by sequencing. Only primers that produced specific and single bands were used for further analysis. qRT–PCR was then performed to quantify the levels of spliced and unspliced transcripts of *MaMADS33*. *MaRPL15* was used as an internal control. The splicing efficiency of individual introns was calculated as the spliced/unspliced ratio^[50]^. Data are presented as the means of three biological replicates, and specific primers used for this analysis are listed in Supplemental Table S1.

### Coding-potential estimation and subcellular localization assay

Coding Potential Calculator 2 (CPC2) was used to analyze the coding potential of various *MaMADS33* isoforms^[51]^. The ORFs of *MaMADS33-AS1* and *MaWAP18* without termination codons were cloned into the pZYGC-GFP vector using the *pEASY*-Basic Seamless Cloning and Assembly Kit (TransGen Biotech, Beijing, China) to produce MaMADS33-AS1-GFP and MaWAP18-GFP fusion constructs driven by the 35S promoter. Primers used are presented in Supplemental Table S1. Plasmids containing the target fragments were then transformed into *Escherichia coli* competent Trans-T1 cells (TransGen Biotech, Beijing, China). The accuracy of the cloned sequences was verified by sequencing. pZYGC-GFP was used as a positive control plasmid. The correctly sequenced plasmids were transformed into *Agrobacterium tumefaciens* strain GV3101 by the freeze-thaw method. GV3101 cells harboring various expression plasmids were then individually transformed into fully expanded leaves of six-week-old *Nicotiana benthamiana* plants as described previously^[52]^. The leaf epidermal cell layers were observed at 2–3 d post-inoculation with an FV1200 laser scanning confocal microscope (Olympus, Tokyo, Japan). At least three independent biological replicates were performed.

### Yeast two-hybrid (Y2H) screen and Y2H assay

Y2H screening was performed with assistance from Oebiotech (Shanghai, China). A normalized cDNA library for Y2H screening was generated by pooling RNA extracted from dormant flower buds, mature mulberry fruits, and flower buds at the floral formation stage collected in December 2016, May 2017, and June 2017, respectively. The Y2H library screen was performed using pGBKT7-MaMADS33-AS4 as the bait in the yeast strain AH109. To further validate the MaMADS33-AS4–MaWAP18 interaction identified in the Y2H screen, a Y2H assay was performed as described previously^[53]^. MaMADS33-AS4-BD served as the bait construct, and MaWAP18-AD served as the prey. pGBKT7-Lam and pGADT7-T were used as negative controls and pGBKT7-53 and pGADT7-T as positive controls. Bait and prey constructs were co-transformed into yeast strain AH109. Transformants were selected on SD-Leu-Trp (SD-LW) medium, gradient-diluted, and plated on SD-Leu-Trp-His-Ade (QX) medium. Pictures were taken after 4 d of incubation at 30 °C and β-galactosidase activity was quantified using the Yeast β-Galactosidase Assay Kit (Thermo Fisher Scientific, MA, USA) according to the manufacturer's instructions. All Y2H experiments were performed with three independent biological replicates. Primers used for this analysis are presented in Supplemental Table S1. Gene annotations were obtained from the Mulberry Genome Database, MorusDB (https://morus.biodb.org/index)^[54]^.

### Bimolecular fluorescence complementation (BiFC) assay

BiFC assays were performed to confirm the protein–protein interaction identified above. The ORFs of MaMADS33-AS4 and MaWAP18 without stop codons were obtained by PCR amplification with gene-specific primers (Supplemental Table S1). MaMADS33-AS4 and MaWAP18 were then independently fused with the N-terminal and C-terminal halves of the yellow fluorescent protein (YFP). A BiFC assay was performed in the leaves of six-week-old *N. benthamiana* plants using the *A. tumefaciens* transformation and injection protocols described above. Leaf epidermal cell layers were observed at 2–3 d post-inoculation with an FV1200 laser scanning confocal microscope (Olympus, Tokyo, Japan). Three independent biological replicates were performed.

### Statistical analysis

Differences between samples were analyzed by one-way analysis of variance (ANOVA) in SPSS version 19 (IBM Corp., Armonk, NY, USA). Means were compared using Duncan's multiple range test (*p* < 0.05).

## Results

### *MaMADS33* expression is positively associated with dormancy in mulberry

**Figure 1:**
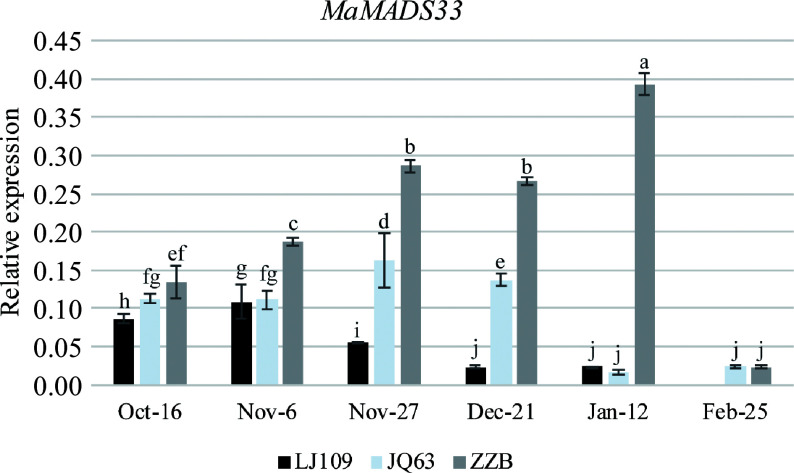
*MaMADS33* expression is positively associated with dormancy in mulberry. *MaMADS33* expression in the mulberry varieties 'Lunjiao109' (LJ109), 'Jinqiang63' (JQ63), and 'Zhenzhubai' (ZZB) was measured throughout dormancy by qRT–PCR using primers targeting the conserved sequence of the MADS-box domain. *MaRPL15* was the internal control gene for normalization of the expression data (*n* = 3; mean ± measurement range). Significant differences are indicated by different lowercase letters (ANOVA and Duncan's multiple range test; *p* < 0.05).

The relationship between *MaMADS33* and dormancy was investigated by comparing *MaMADS33* expression in flower buds from three mulberry varieties with different dormancy durations (LJ109, JQ63, and ZZB) using primers targeting the conserved amino acid sequence of the MADS-box domain (primer I, Supplemental Table S1). *MaMADS33* expression peaked at different times in the three varieties: early November in LJ109, late November in JQ63, and January in ZZB (Fig. 1). Notably, the relative timing of these peaks was consistent with the relative timing of bud break in the three varieties (earliest in LJ109 and latest in ZZB). Expression levels of *MaMADS33* also differed among the mulberry varieties. In October, November, and December, *MaMADS33* expression was highest in ZZB, moderate in JQ63, and lowest in LJ109, again consistent with their relative durations of dormancy. In January, *MaMADS33* expression remained high in ZZB but was low in LJ109 and JQ63. By February, no unopened buds remained on LJ109, and *MaMADS33* expression was low in both JQ63 and ZZB.

### AS affects transcriptions of *MaMADS33* during dormancy

**Figure 2:**
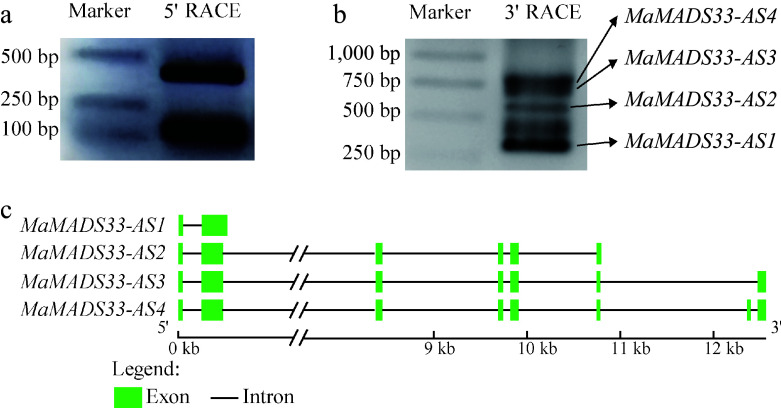
Alternative splicing (AS) of mulberry *MaMADS33* from endodormancy through ecodormancy. (a) 5' RACE, and (b) 3' RACE methods were used to amplify *MaMADS33* transcripts in cDNA pools from JQ63 flower buds collected on five dates from October 2020 through February 2021. Primers were designed to target the conserved sequence of the MADS-box domain. Bands corresponding to *MaMADS33-AS1*, *MaMADS33-AS2*, *MaMADS33-AS3*, and *MaMADS33-AS4* are indicated by arrows. (c) Schematic of *MaMADS33* AS isoforms. Exons are represented by green boxes and introns by lines.

To understand the possible roles of AS during dormancy, *MaMADS33* transcripts were next examined in flower buds of JQ63, which exhibited an intermediate dormancy duration. Using 5' RACE and 3' RACE with specific primers targeting the conserved MADS-box domain sequence (Fig. 2a & b), four distinct complete ORFs of *MaMADS33*: *MaMADS33-AS1*, *MaMADS33-AS2*, *MaMADS33-AS3*, and *MaMADS33-AS4*, with lengths of 327, 555, 633, and 675 bp, respectively were identified. *MaMADS33-AS1* and *MaMADS33-AS2* were truncated isoforms that arose from alternative last exons; *MaMADS33-AS1* terminated in the second intron and *MaMADS33-AS2* in the sixth intron (Fig. 2c). *MaMADS33-AS3* was derived from the skipping of the seventh exon. Finally, *MaMADS33-AS4* represented the complete sequence, including all eight exons.

### *MaMADS33-AS1* is a long non-coding RNA

**Figure 3:**
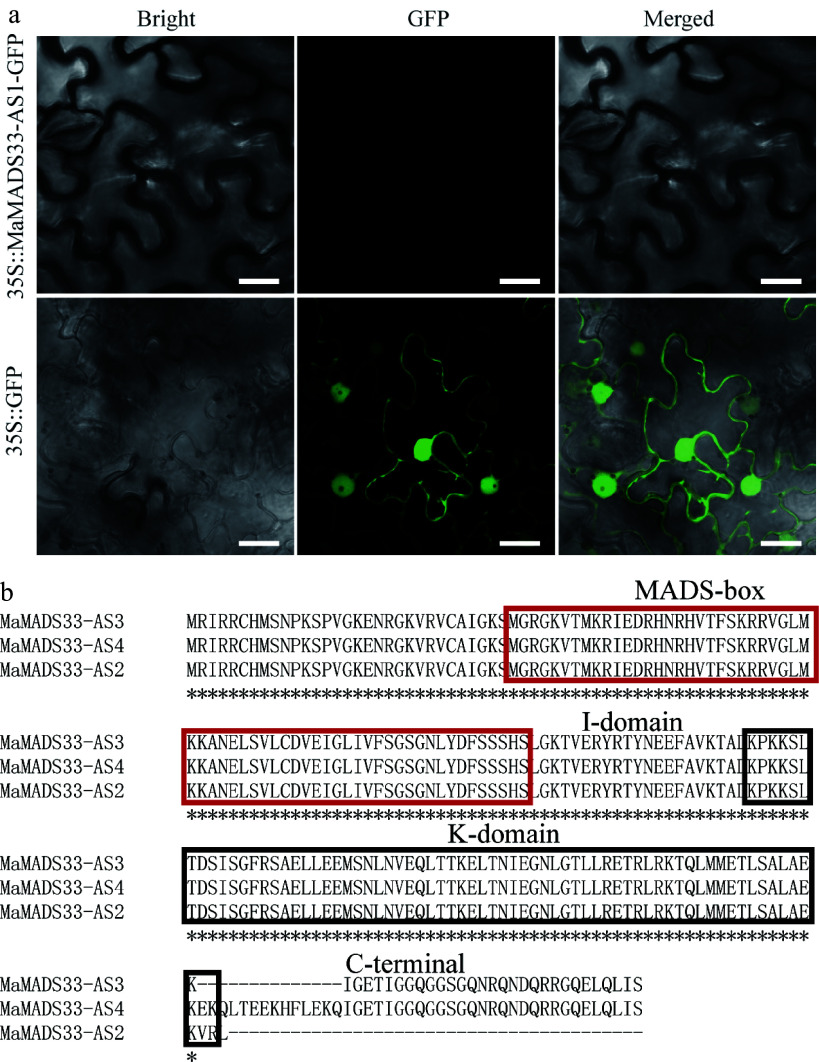
Assessment of *MaMADS33*-*AS1* coding potential and alignment of amino acid sequences. (a) The coding potential of *MaMADS33*-*AS1* was examined by transient expression in *Nicotiana benthamiana* leaves. Scale bar = 20 μm. (b) Amino acid sequences of MaMADS33-AS2, MaMADS33-AS3, and MaMADS33-AS4. The MADS-box and K-domains are indicated by red and black boxes, respectively. The I-domain and C-terminal domain are marked above the sequences.

To investigate the functions of the four *MaMADS33* AS isoforms, their coding potential was examined and it was found that all contained a complete ORF. However, the coding potential of *MaMADS33-AS1* was 0.23, whereas those of *MaMADS33-AS2*, *MaMADS33-AS3*, and *MaMADS33-AS4* were all greater than 0.95. Based on their coding potentials, *MaMADS33-AS1* was classified as a noncoding sequence, and the other three isoforms were classified as coding sequences (Table 1). The low coding potential of *MaMADS33-AS1* was further validated by transient expression of a *MaMADS33-AS1-GFP* construct in *N. benthamiana* leaves, which resulted in no GFP signal (Fig. 3a). These findings confirmed that *MaMADS33-AS1* was a long noncoding RNA.

**Table 1:** Coding potential of the four *MaMADS33* AS isoforms were predicted using Coding Potential Calculator 2 (CPC2).

ID	Label	Coding probability	Peptide length (aa)	ORF integrity
*MaMADS33-AS1*	noncoding	0.232883	109	complete
*MaMADS33-AS2*	coding	0.977475	185	complete
*MaMADS33-AS3*	coding	0.969709	211	complete
*MaMADS33-AS4*	coding	0.985029	225	complete

MaMADS33-AS2, MaMADS3-AS3, and MaMADS03-AS4 were composed of 185, 211, and 225 amino acids, respectively (Table 1). Alignment of their amino acid sequences revealed that MaMADS33-AS2 lacked the C-terminal domain, MaMADS33-AS3 lacked 14 amino acids, mainly in the C-terminal domain, and MaMADS33-AS4 contained all the characteristic domains of MADS family proteins, including the MADS-box, I-domain, K-domain, and C-terminal domain (Fig. 3b).

### *MaMADS33-AS3* and *MaMADS33-AS4* are positively associated with endodormancy

**Figure 4:**
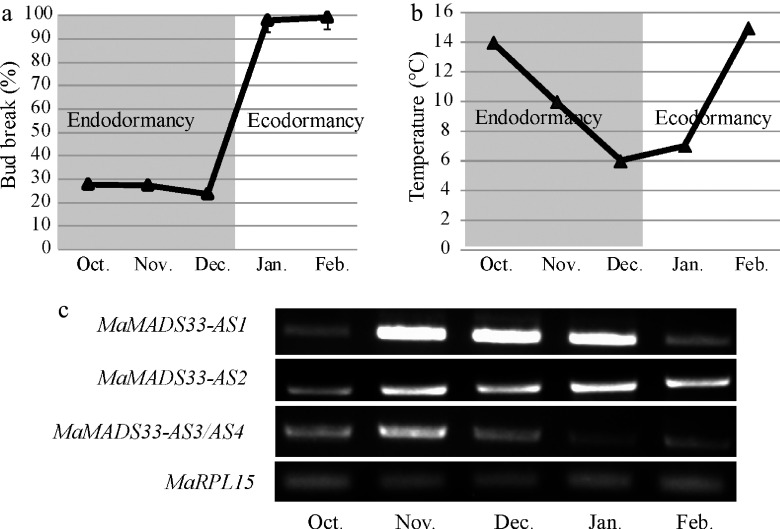
Expression profiles of four *MaMADS33* AS isoforms during dormancy. Dormancy stages of mulberry flower buds. Flower buds of JQ63 were collected from October 2020 through February 2021, and the (a) bud break percentage (*n* = 3, mean ± measurement range), and (b) temperature were recorded. (c) Expression profiles of four *MaMADS33* AS isoforms in flower buds measured by RT–PCR with *MaRPL15* as the reference gene. PCR products were separated on 1% agarose gels.

To investigate the potential functions of *MaMADS33* in endodormancy, the endodormancy stages of JQ63 flower buds were first characterized(Fig. 4a). From October to December, temperatures gradually decreased (Fig. 4b), and the bud-break percentage remained below 50%, indicating that the flower buds were in an endodormant state. Next, the expression of the four *MaMADS33* AS isoforms in bud tissues were measured by RT–PCR with specific primers during endodormancy and ecodormancy (Fig. 4c). Transcript levels of *MaMADS33-AS1* remained low in October, increased significantly from November to December, and then decreased markedly in February. By contrast, expression of *MaMADS33-AS2* increased gradually after October and remained high throughout ecodormancy. Expression of *MaMASD33-AS3* and *MaMADS33-AS4* was relatively high throughout the endodormancy period, peaking around November, then decreased significantly during ecodormancy. To further analyze the expression of these isoforms during endodormancy, nucleic acid bands of *MaMASD33-AS3* and *MaMADS33-AS4* were counted by cloning and sequencing. A total of 44 clones were sequenced, 16 (36.36%) of which were *MaMASD33-AS3* and 28 (63.64%) of which were *MaMADS33-AS4* (Supplemental Table S2). *MaMADS33-AS4* was therefore the predominant isoform in the mixed nucleic acid bands during endodormancy. Expression of these two long protein-coding mRNAs, particularly *MaMADS33-AS4*, thus exhibited a positive association with endodormancy in mulberry flower buds.

### Endodormancy induces AS of *MaMADS33*, resulting in accumulation of *MaMADS33-AS1*

**Figure 5:**
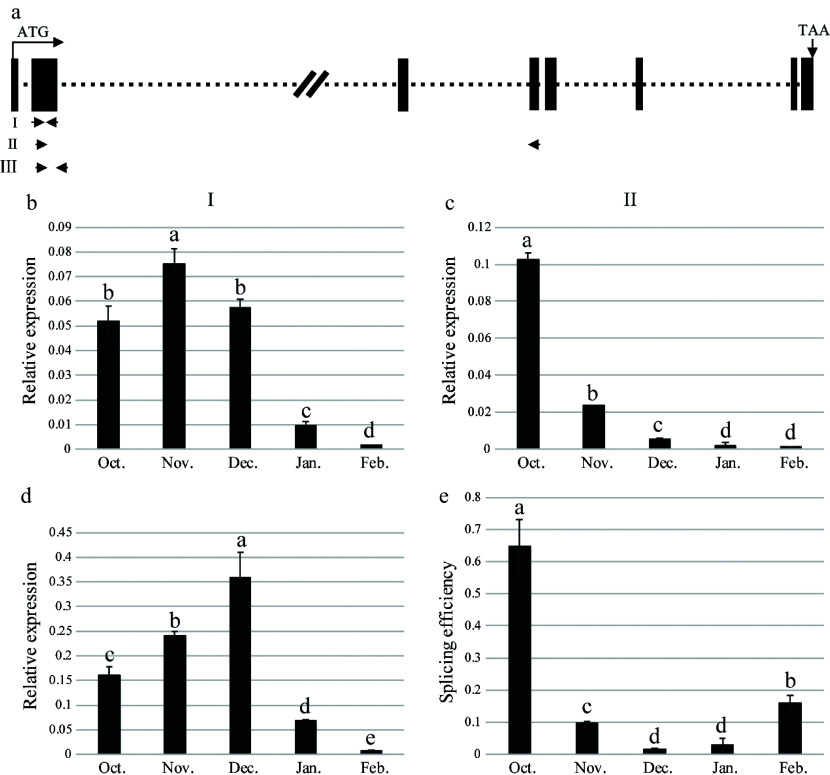
Splicing efficiency of *MaMADS33* intron 2. (a) Schematic of the *MaMADS33* gene. The locations of primers I, II, and III are indicated with arrows. (b) Primer I, located in the conserved MADS-box domain, targeted all spliced and unspliced isoforms of *MaMADS33*. (c) Primer II targeted mRNA from which intron 2 had been spliced, corresponding to transcripts of *MaMADS-AS2*, *MaMADS-AS3*, and *MaMADS-AS4*. (d) Primer III targeted unspliced pre-mRNA for intron 2. (e) Splicing efficiency was calculated as the spliced/unspliced ratio. Relative expression was measured by qRT–PCR using *MaRPL15* as a reference gene (*n* = 3; mean ± measurement range). Flower buds of JQ63 were collected from October 2020 through February 2021. Significant differences are indicated by different lowercase letters (ANOVA and Duncan's multiple range test; *p* < 0.05).

To gain a deeper understanding of the relationship between dormancy and *MaMADS33* expression patterns, the splicing efficiency of intron 2 was analyzed using qRT–PCR data. A schematic representation of the *MaMADS33* gene and the primers used for amplification is provided in Fig. 5a. The amplification products generated by primer I, which targeted the conserved MADS-box domain, corresponded to all *MaMADS33* transcripts (Fig. 5b). Overall transcription of *MaMADS33* was high during endodormancy, then decreased significantly during ecodormancy. The amplification products of primer II, which specifically targeted transcripts from which intron 2 had been spliced, corresponded to *MaMADS33-AS2*, *MaMADS33-AS3*, and *MaMADS33-AS4*. Notably, the abundance of these spliced transcripts decreased after October (Fig. 5c). The amplification products generated by primer III represented unspliced pre-mRNA containing intron 2 (Fig. 5d). To quantify the splicing efficiency of intron 2 in the isoforms *MaMADS33-AS2*, *MaMADS33-AS3*, and *MaMADS33-AS4*, the spliced/unspliced ratio was calculated based on qRT–PCR data obtained with primer II vs primer III. The results revealed a significant decrease in splicing efficiency during endodormancy, followed by a gradual increase during ecodormancy (Fig. 5e).

### MaMADS33-AS4 interacts with MaWAP18 during endodormancy

**Figure 6:**
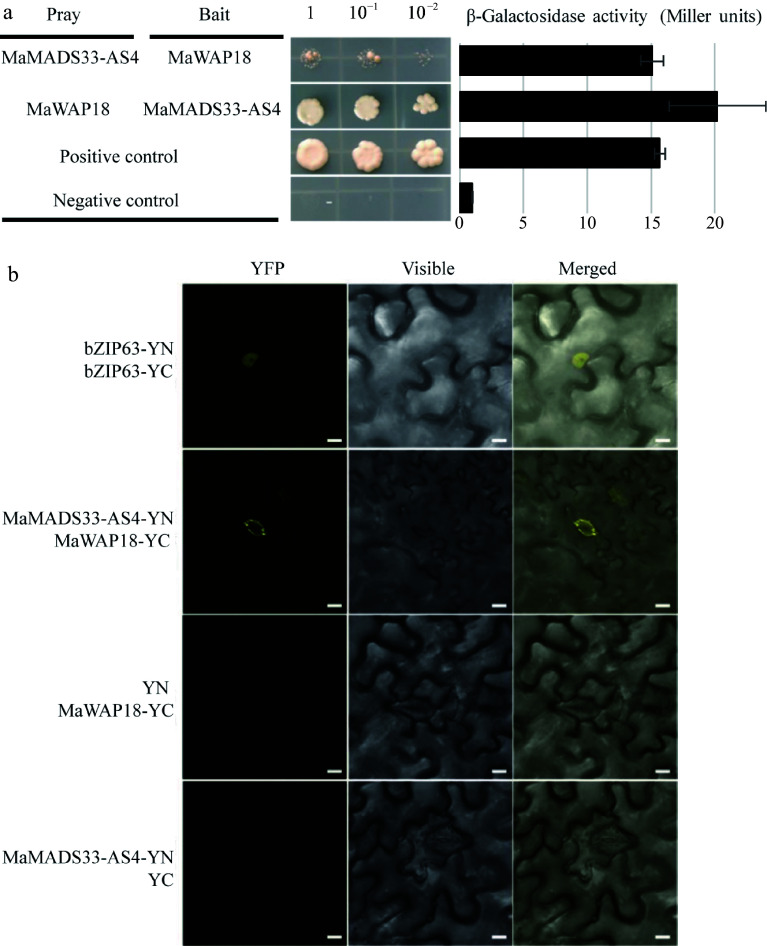
Interaction between MaMADS33-AS4 and MaWAP18. (a) Interaction of MaMADS33-AS4 and MaWAP18 in yeast. pGBKT7-p53 was mated with pGADT7-T as a positive control, and pGBKT7-Lam was mated with pGADT7-T as a negative control. Yeast was diluted 1, 10, and 100 fold before plating onto quadruple dropout (QDO) medium. Corresponding measurements of β-galactosidase activity are shown. Three independent experiments were performed with similar results. (b) BiFC assay in 4-week-old *Agrobacterium*-infiltrated *N. benthamiana* leaves. MaMADS33-AS4 and MaWAP18 were independently fused to the N-terminal and C-terminal halves of yellow fluorescent protein (YFP), respectively. Images of YFP fluorescence were obtained using a confocal microscope. Scale bar = 10 μm. Three independent experiments were performed with similar results.

**Figure 7:**
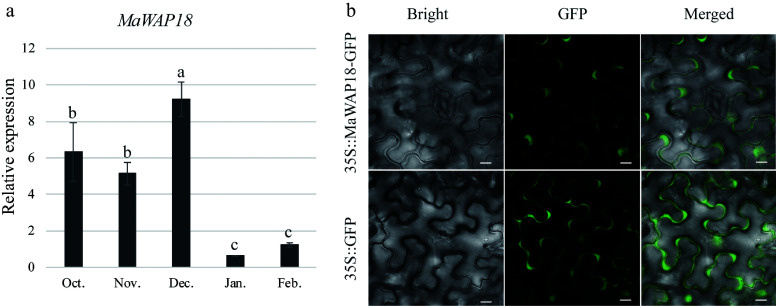
Expression and subcellular localization analyses of MaWAP18. (a) Relative expression was measured by qRT–PCR using *MaRPL15* as the reference gene (*n* = 3; mean ± measurement range). Flower buds of JQ63 were collected from October 2020 through February 2021. (b) Subcellular localization of MaWAP18. Images of 4-week-old *Agrobacterium*-infiltrated *N*. *benthamiana* leaves expressing the MaWAP18-GFP fusion protein driven by the CaMV 35S promoter were obtained under green fluorescence, merged light, and visible light. 35S:GFP was used as a positive control. Scale bar = 10 μm. Significant differences are indicated by different lowercase letters (ANOVA and Duncan's multiple range test; *p* < 0.05).

To further investigate the potential mechanism by which MaMADS33 functions in endodormancy, a Y2H screen of pooled RNA from dormant flower buds, mature fruits, and flower buds at the floral formation stage was performed. All interacting proteins identified are listed in Supplemental Table S3; among them was the winter-accumulating protein MaWAP18. We next performed a Y2H and β-galactosidase activity assay to further examine the interaction between MaMADS33-AS4 and MaWAP18 (Fig. 6a). The interaction between MaMADS33-AS4 and MaWAP18 was strong when MaMADS33-AS4 served as the bait and MaWAP18 as the prey. To confirm this interaction *in vivo*, a BiFC assay in *N. benthamiana* was performed. Transient co-expression of MaMADS33-AS4-YFPN and MaWAP18-YFPC in tobacco leaves resulted in a distinct yellow fluorescence signal in the guard cells, conclusively demonstrating the interaction between MaMADS33-AS4 and MaWAP18 *in planta* (Fig. 6b). To determine whether the expression of MaWAP18 was also associated with endodormancy, its expression profile was analyzed through time. Expression of *MaWAP18* was high during endodormancy and low during ecodormancy (Fig. 7a). Subcellular localization analysis suggested that MaWAP18 was localized primarily in the cell membrane and nucleus, although it was also detected in the guard cells of *N. benthamiana* leaves (Fig. 7b).

## Discussion

### *FLC* homologs are involved in endodormancy

Endodormancy of woody plants and vernalization of annual plants involve similar biological processes induced by cold or light, including growth cessation, slowed development or stagnation, and release, enabling plants to survive adverse winter conditions^[55,56]^. The duration of effective cold exposure during winter has a direct effect on the release from endodormancy and the vernalization process^[57,58]^. Some researchers have reported that endodormancy and vernalization share common mechanisms^[59−61]^. During the vernalization process, the regulation of the *FLC* gene can be divided into several stages. Before vernalization, *FLC* is activated to establish the conditions necessary for vernalization. As vernalization proceeds, expression of the long noncoding RNAs *COOLAIR* and *COLDAIR* suppresses *FLC* expression. Changes in chromosome conformation also contribute to the decrease in *FLC* expression. Finally, after vernalization and before the floral transition, epigenetic modifications and high expression of *VERNALIZATION INSENSITIVE 3* (*VIN3*) maintain *FLC* in a silenced state^[26,62]^.

*FLC* is also involved in *Arabidopsis* seed dormancy through mechanisms similar to those of vernalization^[63−65]^, and the *MdoFLC* gene in apple co-localizes with a bud-break QTL^[29]^. Previous studies in perennial plants have reported upregulation of *FLC* genes during endodormancy and downregulation during ecodormancy; examples include *PtFLC* in trifoliate orange^[27]^, *MdoFLC* in apple^[29]^, and *CsFLC1* in *Camellia sinensis*^[66]^. Recently, similar expression patterns were documented for *MnMADS33* (i.e., *MaMADS33-AS4*) in mulberry using primers that targeted conserved domain sequences^[43]^. However, previous expression profiles were based solely on quantitative detection using single primers and may therefore not accurately reflect gene expression, especially for genes with AS. Therefore, in this study, *MaMADS33* expression in mulberry was systematically analyzed using both qRT–PCR and RT–PCR with multiple primers. Combined with measurements of mulberry endodormancy, these expression data indicated that *MaMADS33* expression was positively associated with endodormancy of flower buds, and this was particularly true for the longer *MaMADS33-AS3* and *MaMADS33-AS4* isoforms. The long noncoding RNA *MaMADS33-AS1* was also identified, which accumulated during the late stage of endodormancy and in the early stage of ecodormancy. These findings further support the proposed role of *FLC* homologs as important regulators of endodormancy.

### Chilling promotes AS of *MaMADS33* during endodormancy

A brief period of chilling in autumn triggers the onset of bud dormancy in apple, and a longer duration of chilling ultimately results in dormancy release^[67]^. Low temperatures are known to trigger extensive and rapid alterations in the RNA isoforms produced in response to environmental fluctuations in *Arabidopsis*^[68]^. For the mulberry variety JQ63, temperatures gradually decreased during endodormancy and rose during ecodormancy (Fig. 4a), and the splicing efficiency of *MaMADS33* intron 2 increased with increasing temperature (Figs 4b & 5e). Previous studies of *Arabidopsis* have shown that the majority of cold-regulated AS events introduce premature termination codons into transcripts^[69]^. Consistent with this observation, *MaMADS33-AS1* and *MaMADS33-AS2* were truncated versions of *MaMADS33* that arose from alternative last exons. We therefore suggest that chilling temperatures induce AS of *MaMADS33*, resulting in the production of shorter isoforms, particularly *MaMADS33-AS1*. However, further study will be required to determine the role and mechanism by which *MaMADS33-AS1* participates in dormancy.

### Interaction between MaMADS33-AS4 and MaWAP18

Pathogenesis-related protein 10 (PR10) plays a crucial role in plant responses to biotic and abiotic stresses^[70−72]^. MaWAP18, a PR10 homolog accumulates during winter in mulberry (*Morus bombycis* Koidz.)^[73]^, and purified WAP18 from *M. bombycis* exhibits cryoprotective activity towards lactate dehydrogenase *in vitro*^[73]^, suggesting that it has a role in freezing tolerance. A similar pattern of PR10-like protein accumulation during dormancy was reported in *Retama raetam*^[74]^, and cold acclimation triggered the accumulation of a PR10 homolog in white pine^[75]^. It was also found that the expression of *MaWAP18* was high during endodormancy (Fig. 7a). The subcellular localization assay demonstrated that MaWAP18 was localized to the cell membrane, nucleus, and guard cells in *N. benthamiana* leaves, displaying a localization pattern similar to that observed for MaMADS33-AS4^[43]^. Notably, an interaction between MaMADS33-AS4 and MaWAP18 was observed in stomata (Fig. 6b). Low temperatures are known to promote ABA accumulation, leading to stomatal closure^[76]^, and studies have shown that application of exogenous ABA can improve plant cold tolerance^[77]^. In our previous report, *MaMADS33-AS4* expression was upregulated by both exogenous ABA and cold treatment^[43]^. Taken together, these findings suggest that MaMADS33-AS4, induced by ABA, may interact with MaWAP18 at the stomata of flower buds, thereby contributing to cold tolerance, although this possibility remains to be tested.

## Conclusions

Four isoforms of *MaMADS33* resulting from AS were identified. Expression profiling revealed that the overall abundance of *MaMADS33* transcripts increased during endodormancy. Expression levels of *MaMADS33-AS3* and *MaMADS33-AS4* showed a positive association with endodormancy in flower buds. The splicing efficiency of *MaMADS33* intron 2 decreased during endodormancy, leading to the accumulation of the truncated long noncoding RNA *MaMADS33-AS1*. Furthermore, MaWAP18, which has been suggested to play a role in the acquisition of freezing tolerance, was identified as an interaction partner of MaMADS33-AS4. These findings shed light on the molecular mechanisms of endodormancy in woody plants and contribute to a better understanding of the roles of *FLC* homologs in endodormancy.

## SUPPLEMENTARY DATA

Supplementary data to this article can be found online.

## Data Availability

The datasets generated during and/or analyzed during the current study are available from the corresponding author on reasonable request.
